# Recurring Carcinoma of the Breast, Injections of Quinine Bisulphate Rendered Radio-Active by Roentgen Rays

**Published:** 1914-06

**Authors:** 


**Recurring Carcinoma of the Breast, Injections of Quinine Bisulphate Rendered Radio-Active by Roentgen Rays.** Max Reichmann, of Chicago, Ill., Medical Journal, April, 1914. Healing of ulceration, freer movement of arm, disappearance of tumors were noted.

